# A Text-Book of Materia Medica and Therapeutics

**Published:** 1903-11

**Authors:** 


A Text-Book of Materia Medica and Therapeutics.—By A. A. Stevens, A. M., M. D., Lecturer on Physical Diagnosis in the University of Pennsylvania; Physician to the Episcopal and St. Agnes Hospitals, Philadelphia. Third edition, greatly enlarged, rewritten and reset. Handsome octavo of 663 pages. W. B. Saunders & Company. 1903. Cloth, $3.50 net.

The arrangement of the work throughout is a very systematic one. The drugs are considered, not alphabetically, as in many textbooks, but according to their pharmacologic action, which gives to the book a sort of correlative value, and is thus of great help to the student. Following the general description of each drug is given briefly its therapeutic indications, but the practical use of the drug is for the most part discussed under the heading of the disease or symptom for which it is most often prescribed, almost a third of the work being devoted to the discussion of applied therapeutics.

Electricity, mechanical therapeutics, etc., together with the newer remedies are thoroughly and briefly discussed, making the book on the whole a very practical one. J. M. L.

